# Appropriate Selection of the Initial Diagnostic Catheter for Left Coronary Angiography Using Computed Tomography

**DOI:** 10.7759/cureus.75004

**Published:** 2024-12-02

**Authors:** Tatsuhiro Fujimura, Masaki Takemitsu, Reina Murayama, Taisei Nishimura, Yosuke Miyazaki, Tetsuya Matsuyama, Yuki Nakata, Takayuki Okamura, Motoaki Sano

**Affiliations:** 1 Division of Cardiology, Department of Medicine and Clinical Science, Graduate School of Medicine, Yamaguchi University, Ube, JPN; 2 Department of Radiological Technology, Yamaguchi University Hospital, Ube, JPN

**Keywords:** aortic morphology, computed tomography, coronary angiography, coronary diagnostic catheters, left coronary artery

## Abstract

Purpose: In patients with a dilated ascending aorta, a diagnostic catheter with a larger curve than the Judkins left 4.0 (JL4) is occasionally required to engage the left coronary artery. However, the specific size of the ascending aorta and other parameters have not been sufficiently investigated. We examined the relationship between aortic morphological parameters and the need for a larger catheter size during left coronary angiography (CAG).

Methods: At our hospital, consecutive patients who underwent both CAG and contrast-enhanced computed tomography (CT) for aorta and coronary imaging were divided into two groups based on the catheter used for left CAG: the JL4 group and the Judkins left 5.0 (JL5) group. Nine selected aortic morphological parameters from the CT images were measured and compared between the two groups.

Results: The JL5 and JL4 groups included 19 and 230 patients, respectively. The JL5 group had higher numbers of coronary diagnostic catheters used, longer procedure times, and greater contrast volumes compared to the JL4 group. Among the nine aortic morphological parameters, significant differences were found in the maximum area and length of the ascending aorta, the total length of the aorta, and the width and depth of the aorta. Multivariate analysis revealed that the maximum area of the ascending aorta was most strongly associated with the need for JL5 in left CAG (odds ratio (95% CI) per 100 mm^2^, 1.71 (1.33-2.21), p < 0.0001). The cutoff value for the maximum area of the ascending aorta was 1111.2 mm^2^ (corresponding to an ascending aortic diameter of approximately 38 mm).

Conclusion: Selecting a large-curve diagnostic catheter, such as the JL5, as the initial diagnostic catheter for engaging the left coronary artery in patients with an ascending aorta diameter greater than 38 mm on CT may optimize left CAG.

## Introduction

Coronary angiography (CAG) remains the gold standard for defining coronary anatomy and assessing the severity of coronary arterial stenoses [1]. The Judkins left 4.0 (JL4) diagnostic catheter allows entry into the left coronary ostium with minimal manipulation in 80%-90% of patients [2]. However, in patients with a dilated ascending aorta, larger-curved diagnostic catheters, such as the Judkins left 5.0 (JL5) and Judkins left 6.0 (JL6), are occasionally required [3]. Several complications of CAG tests, such as contrast-induced nephropathy, arterial dissection, and cerebral or myocardial embolization, remain. Appropriate selection of the initial diagnostic catheter could reduce the amount of contrast usage, unnecessary catheter manipulation, procedure time, and the risk of dissection and embolization.

Coronary computed tomography (CT) angiography has proven useful for evaluating coronary arteries. It is frequently performed before CAG because it is a noninvasive technique with a low risk of major complications. However, its widespread use has reduced opportunities for young interventionalists to gain experience performing invasive CAG [4,5]. In this era of limited CAG opportunities, obtaining meaningful information, such as aortic morphology that requires a larger diagnostic coronary catheter before angiography, could help optimize the procedure, even for less-experienced operators.

We investigated the aortic morphological features on CT in patients who required a larger-curved diagnostic catheter (JL5) to engage the left coronary artery, as current guidelines and practices do not clearly define the cutoff values for ascending aorta size and other parameters that would indicate its use.

## Materials and methods

Patients who underwent CAG and CT of the aorta and coronary arteries at Yamaguchi University Hospital from January 1, 2015, to August 31, 2020, were retrospectively identified. The patients were divided into JL4 and JL5 groups based on the catheter ultimately used to engage the left coronary artery (JL6 was not used in our hospital). All diagnostic catheters used were 5 Fr in size.

Exclusion criteria included patients who underwent simultaneously coronary artery bypass angiography, those who received treatments for aortic artery or left coronary artery ostium between the two tests, those with more than six months between the two tests due to potential annual changes in the aorta, those who used Judkins left 3.5 or a single catheter to focus on the use of JL5, and those with poor-quality images that could not be assessed by automatic measurement software on CT.

Procedure characteristics

Approach sites were categorized as radial, femoral, and brachial arteries. The left- and right-upper-limb approaches referred to the left and right radial and brachial artery approaches, based on the different catheter insertion angles into the ascending aorta. Procedure time and contrast volume used exclusively for CAG were recorded. The years of experience of the operator were also considered to account for potential bias.

CT image acquisition

Coronary and aorta images were acquired using a dual-source CT scanner (SOMATOM Force, Siemens Healthcare, Erlangen, Germany). To assess the coronary arteries and aortic valve, a retrospective electrocardiogram (ECG)-triggered helical scan was performed with a detector collimation of 2 × 96 × 0.6 mm, a rotation time of 0.25 seconds, and a pitch factor of 0.15-0.34, automatically adjusted to the patient’s heart rate. Immediately following, a high-pitch, non-ECG-gated scan was conducted to evaluate the entire aorta, minimizing motion artifacts. The non-ECG-gated scan parameters include a detector collimation of 2 × 96 × 0.6 mm, a rotation time of 0.25 seconds, and a pitch factor of 1.55. Based on the patient's weight, 70-90 mL of contrast agent (Iopamiro 370, iopamidol, Bayer Healthcare, Tokyo, Japan; or Omnipaque 350, iohexol, GE HealthCare Pharma, Tokyo, Japan) was injected at a flow rate of 4-5.5 mL/second, followed by a 30-mL saline flash at the same flow rate. Moreover, the contrast volume was determined based on body weight and the imaged areas. 

Aortic morphological parameters in CT

Aortic morphological parameters were analyzed using Ziostation (AMIN Inc, Tokyo, Japan). Nine aortic and coronary morphological parameters were assessed from the CT images: (1) aortic angle, measured as the angle between the horizontal line and the aortic centerline in front of the maximum-intensity projection (MIP) image; (2) right coronary ostium angle, measured relative to the 3 o’clock position in a perpendicular image reconstructed for preoperative transcatheter aortic valve implantation assessment [6]; (3) left coronary ostium angle, measured similarly to the right coronary ostium angle; (4) maximum area of the ascending aorta, automatically measured using a curved multiplanar reconstruction (MPR) view that detects the aorta centerline; (5) ascending aortic length, defined as the length from the Valsalva sinus to the origin of the brachiocephalic artery, measured on the same curved MPR view; (6) total length of the aorta, defined as the length from the Valsalva sinus to the bifurcation carina of the common iliac arteries; (7) height of the aorta, measured from the front of the MIP image of the aorta; (8) width of the aorta, measured from the side of the MIP image of the aorta; and (9) depth of the aorta, also measured from the side of the MIP image of the aorta.

These measurements were conducted by three CT imaging specialists (T.F., M.T., and R.M.) and compared between the JL4 and JL5 groups.

Statistical analyses

Continuous variables are presented as medians with first and third interquartile ranges (IQRs) and were compared using the nonparametric Wilcoxon rank-sum test for non-normally distributed variables. Categorical variables are presented as frequencies (proportions) and were compared using the χ^2^ test or Fisher's exact test. Multivariable predictors for the need for JL5 in left CAG were identified using stepwise logistic regression analysis and are presented as odds ratios with 95% CIs. A p < 0.05 was considered statistically significant.

To assess the reproducibility of the nine aortic morphological measurements on CT, the intraclass correlation coefficient was calculated from 30 randomly selected cases. The Evaluating the Measurement Process (EMP) method for measurement system analysis was used, as the measurements were evaluated by three researchers (T.F., M.T., and R.M.). Statistical analyses were performed using JMP for Windows, Version 16 (SAS Institute, Cary, NC, USA).

## Results

Study population, lesion characteristics, and procedure details

**Figure 1:**
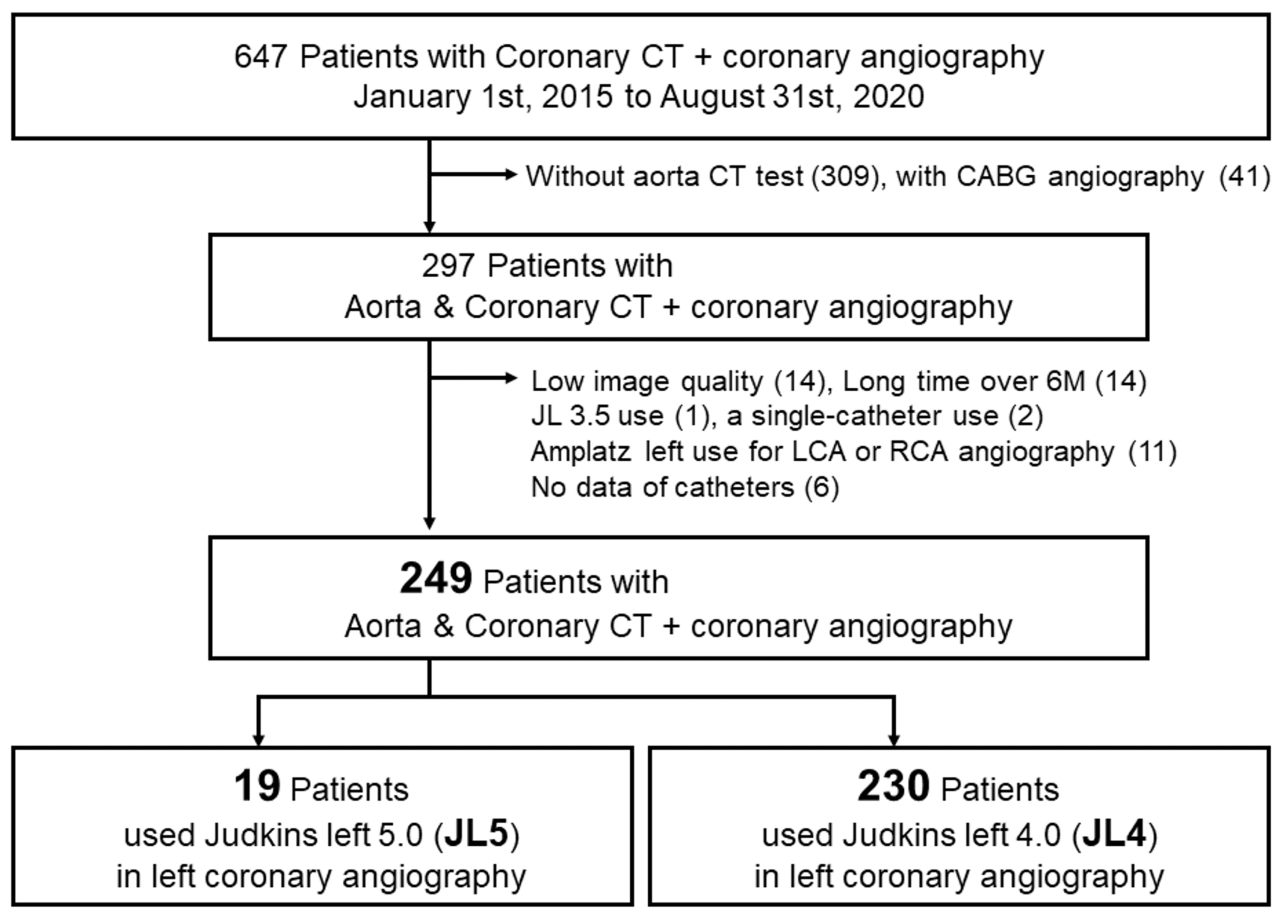
Flowchart illustrating the study population, showing the distribution of patients in the JL5 (19 patients) and JL4 (230 patients) groups CABG: coronary artery bypass graft, CT: computed tomography, LCA: left coronary artery, RCA: right coronary artery.

Of the 647 patients who underwent coronary CT (n = 3643) and CAG (n = 2865), 249 had assessable aorta and coronary CT images. The JL5 and JL4 groups consisted of 19 and 230 patients, respectively (Figure 1). Significant differences were noted in body height, age, smoking, and the prevalence of peripheral artery disease. In the JL5 group, 12 patients underwent CAG exclusively with the JL5 catheter based on accumulated CAG information and operator discretion, while JL5 was used after initially attempting JL4 in seven patients. Amplatz left 1.0 or 2.0 catheters were utilized for right CAG in 18 patients from the JL5 group. The JL5 group required more catheters, longer procedure time, and greater contrast volumes for CAG compared to the JL4 group. There was no significant difference in operator experience between the two groups (Table 1).

**Table 1:** Patient demographics and procedure characteristics Values are presented as percentages (n) or median and IQRs. Five cases in the JL4 with a history of post-CABG did not undergo bypass graft angiography. CABG: coronary artery bypass graft, COPD: chronic obstructive pulmonary disease, PCI: percutaneous coronary intervention, RCA angio: angiography for right coronary artery.

	JL5 (n = 19)	JL4 (n = 230)	p-value
Age, year	69 (64, 74)	79 (72.0, 84.0)	0.0013
Body height, cm	161.5 (154, 166)	154.5 (145.6, 163)	0.01
Body weight, kg	63.1 (55.0, 70.8)	54 (44.5, 63.0)	0.0021
Body mass index, kg/m^2^	23.8 (22.3, 26.3)	22.4 (20.0, 25.1)	0.0473
Male, n (%)	14 (73.7)	120 (52.2)	0.07
Hypertension, n (%)	14 (73.7)	187 (81.3)	0.42
Hyperlipidemia, n (%)	13 (68.4)	152 (66.1)	0.84
Diabetes mellitus, n (%)	3 (15.8)	85 (36.9)	0.05
Smoking, n (%)	13 (68.4)	97 (42.2)	0.03
Post-PCI, n (%)	2 (10.5)	30 (13.0)	0.75
Post-CABG, n (%)	0 (0.0)	5 (2.2)	0.52
Cerebrovascular disease, n (%)	1 (5.26)	56 (24.4)	0.06
Peripheral artery disease, n (%)	2 (10.5)	47 (20.4)	0.3
COPD, n (%)	4 (21.1)	42 (18.3)	0.76
Chronic kidney disease, n (%)	3 (15.8)	47 (20.4)	0.63
Severe aortic stenosis, n (%)	12 (63.2)	148 (64.4)	0.92
Ischemic heart disease, n (%)	8 (42.1)	94 (40.9)	0.92
Approach site, n (%)			0.61
Radial artery	16 (84.2)	178 (77.4)	
Femoral artery	3 (15.8)	42 (18.3)	
Brachial artery	0 (0.0)	10 (4.4)	
Diagnostic catheters, n (%)			<0.0001
Judkins left 5.0	19 (100.0)	0 (0.0)	
Judkins left 4.0	7 (36.8)	230 (100.0)	
Judkins right 4.0	18 (94.7)	230 (100.0)	
Amplatz 1.0 or 2.0 for RCA angio	6 (31.6)	0 (0.0)	
Total, n (/patient)	50 (2.6)	460 (2.0)	
Years of experience as an operator, year	4.0 (2.0, 13)	3.5 (0, 5.8)	0.13
During the time between the two tests, day	5.0 (2.0, 10.0)	6.0 (3.0, 18.3)	0.10
Procedure time, min	23 (18, 32)	13 (6, 19.5)	0.0004
Contrast volume, mL	55 (40, 71.3)	40 (30, 50)	0.01

Aortic morphological findings requiring other coronary catheters

**Figure 2:**
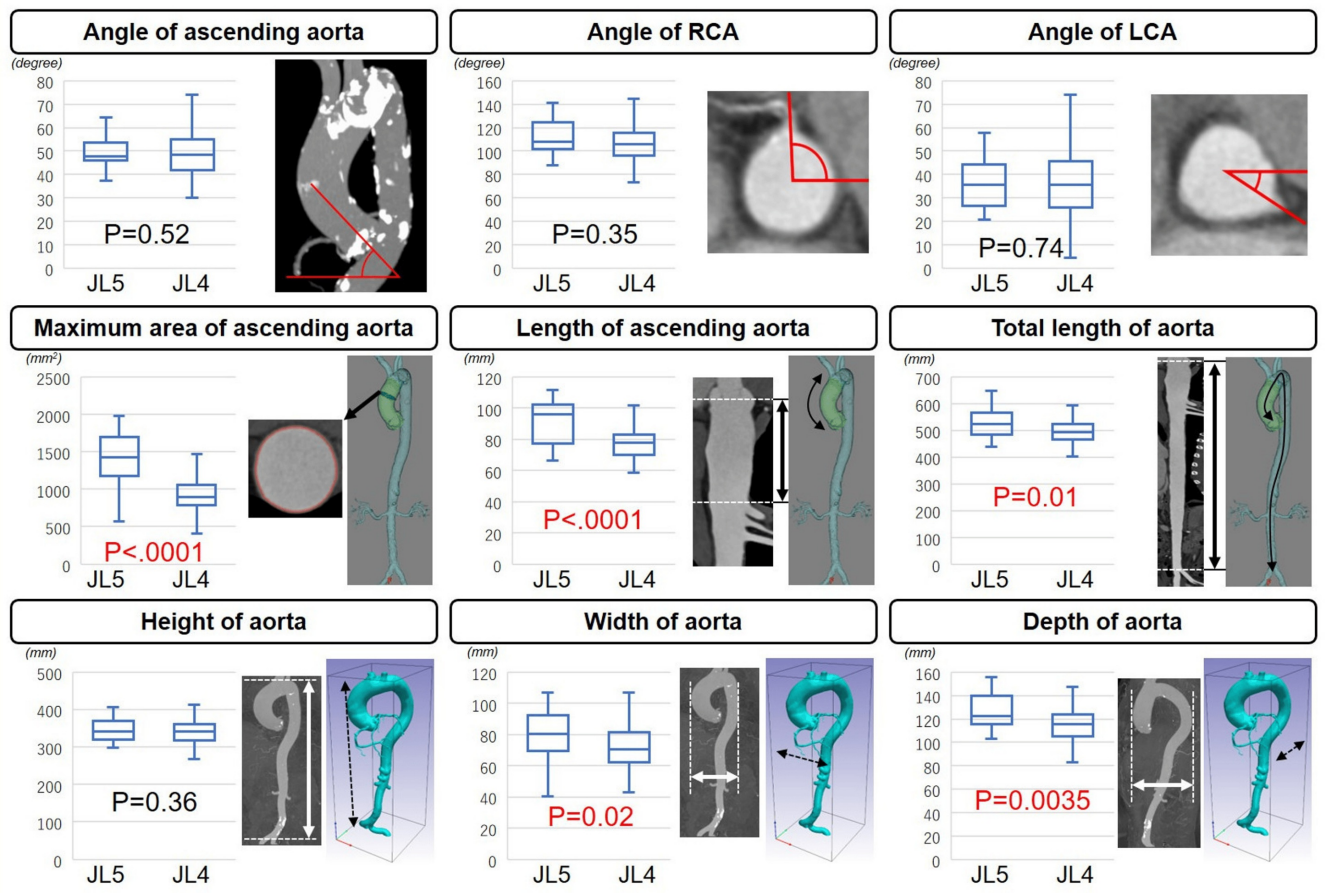
Differences in nine morphological findings between the JL5 group and JL4 group The red numbers indicate significant differences. JL4: Judkins left 4.0 group, JL5: Judkins left 5.0 group, LCA: left coronary artery, RCA: right coronary artery.

The maximum area of the ascending aorta was larger in the JL5 group compared to the JL4 group (JL5, 1425 mm^2^ (1179, 1701); JL4, 898 mm^2^ (783, 1062); p < 0.0001). Additionally, the length of the ascending aorta (JL5, 96 mm (78, 103); JL4, 78 mm (70, 83); p = 0.0001) and the total length of the aorta (JL5, 525 mm (486, 566); 494 mm (466, 522); p = 0.01) were greater in the JL5 than in the JL4 group. The width (JL5, 80 mm (69, 92); JL4, 71 mm (62, 81); p = 0.02) and depth (JL5, 122 mm (115, 140); JL4, 115 mm (105, 124), p = 0.0035) of the aorta were also greater in the JL5 group compared to the JL4 group (Figure 2; Supplemental material 1).

In the multivariate analysis of aortic morphological parameters, the angle of the RCA, the maximum area of the ascending aorta, the length of the ascending aorta, and the width of the aorta were included in a stepwise analysis. Ultimately, the maximum area of the ascending aorta was found to be most strongly associated with the use of a larger-curved coronary catheter in the logistic analysis (Table 2). Regarding the approach site, significant differences were observed in the maximum area, length, and width of the ascending aorta in the left-upper-limb-approach group. In the right-upper-limb approach group, significant differences were noted for the maximum area, length, total length, width, and height of the ascending aorta. No significant differences in aortic morphological parameters were observed in the femoral-artery-approach group (Supplemental material 1). The reproducibility of the nine aortic morphological parameters exceeded a reliability score of 0.8 in all tests (Supplemental material 2).

**Table 2:** Univariate and multivariate analyses of each aortic finding associated with the need for a JL5 catheter in left coronary angiography The odds ratios were calculated as the ratio of 10 changes for all items except for the maximum area of the ascending aorta (of 100 changes). CI: confidence interval, LCA: left coronary artery, RCA: right coronary artery.

	Univariate analysis			Multivariate logistic analysis		
	Odds ratio	95% CI	p-value	Odds ratio	95% CI	p-value
Angle of the ascending aorta, degrees	1.15	(0.70-1.88)	0.57	-	-	-
Angle of the RCA, degrees	1.18	(0.87-1.62)	0.27	1.73	(1.03-2.92)	0.04
Angle of the LCA, degrees	0.98	(0.73-1.30)	0.87	-	-	-
Maximum area of the ascending aorta, mm^2^	1.63	(1.36-1.95)	<0.0001	1.71	(1.33-2.21)	<0.0001
Length of the ascending aorta, mm	3.09	(1.95-4.91)	<0.0001	4.79	(2.09-10.9)	0.0002
Total length of the aorta, mm	1.16	(1.04-1.28)	0.0055	-	-	-
Width of the aorta, mm	1.10	(0.94-1.28)	0.26	-	-	-
Height of the aorta, mm	1.40	(1.04-1.88)	0.03	0.54	(0.30-0.95)	0.03
Depth of the aorta, mm	1.95	(1.32-2.87)	0.0007	-	-	-

**Figure 3:**
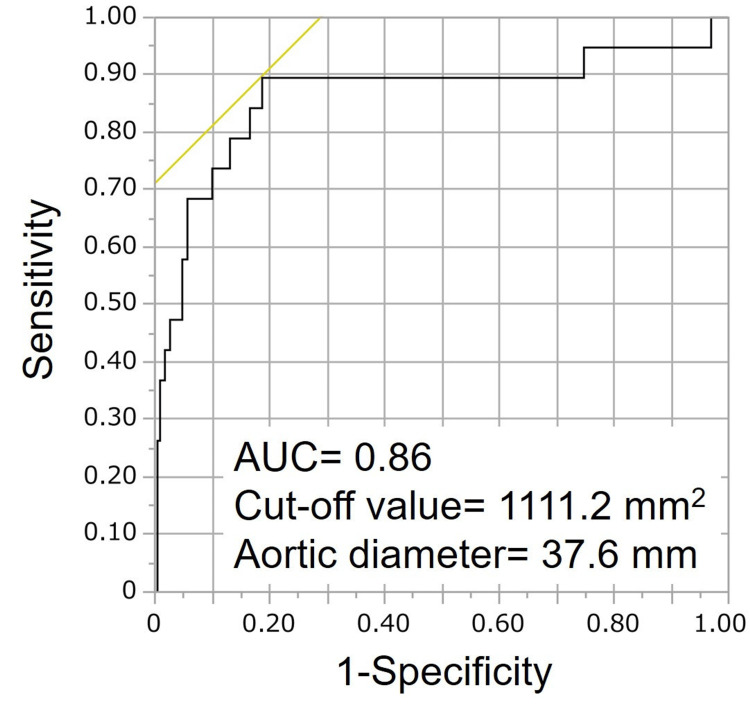
Receiver operating characteristic curve of the maximum area of the ascending aorta using JL5 catheter in left coronary angiography JL5: Judkins left 5.0.

The maximum area of the ascending aorta was emphasized due to its relevance in multivariate analysis and its clinical applicability. The cutoff value and the area under the curve (AUC) for the receiver operating characteristic curve of the maximum area of the ascending aorta in patients requiring a larger-curved coronary catheter were 1111.2 mm^2^ and 0.86, respectively. The corresponding aortic diameter derived from the cutoff value was 37.6 mm (Figure 3). In the left-upper-limb-approach and right-upper-limb-approach groups, which showed significantly different maximum areas of the ascending aorta, the cutoff values and aortic diameters were similar to those obtained from the overall data. The values were 1270.4 mm^2^ (aortic diameter = 40.2 mm) for the left-upper-limb approach and 1147.1 mm^2^ (aortic diameter = 38.2mm) for the right-upper-limb approach (Supplemental material 3). Moreover, the average aortic diameter, derived from both the long and short diameters at the maximum area of the ascending aorta, was also associated with the use of a JL5 catheter. The corresponding cutoff value and AUC for this measurement were 0.83 and 38.6 mm, respectively (Supplemental material 4).

## Discussion

This study demonstrated that the maximum area of the ascending aorta was the most significant factor associated with the use of a JL5 catheter for left CAG among the nine aortic morphological findings on CT. The cutoff value for the maximum area of the ascending aorta was 1111.2 mm^2^, with the derived diameter being 37.6 mm. The JL5 group required more catheters, longer procedure times, and greater contrast volumes for CAG compared to the JL4 group.

The Judkins catheter technique is easy to learn, allowing for rapid and consistent selective coronary catheterization [7]. The Judkins right 4.0 (JR4) and JL4 catheters are commonly used as the first choice for CAG worldwide. However, there are instances when engaging the left coronary artery with the JL4 catheter is challenging. While it is well established that factors such as dilation of the ascending aortic root, the angle of the ascending aorta, elongation of the aorta, and rotation of the aorta play significant roles [2], the precise size of the ascending aorta and the key factors indicating the use of the JL5 for left CAG have not been sufficiently investigated. Coronary CT angiography is performed prior to CAG due to its noninvasive nature and its ability to provide valuable data. Recently, opportunities for young interventionalists to gain experience in performing invasive CAG have diminished, making it essential to gather information beforehand to optimize the procedure. We investigated the factors related to aortic morphology on CT that necessitate the use of larger-curved diagnostic catheters in left CAG, as these details have not been clearly defined. Our findings suggest that a cutoff value of 1111.2 mm^2^ for the maximum area of the ascending aorta (derived diameter, 37.6 mm) is reasonable, considering that the JL4 catheter has a curve of 4 cm. Based on these results, routinely using the JL5 for first-attempt left CAG in cases with ascending aortas dilated to over approximately 38 mm on preliminary CT could potentially reduce costs, procedure time, and contrast volume. These improvements could lead to a reduction in complications of CAG, such as contrast-induced nephropathy, arterial dissection, and unexpected embolism.

The aorta enlarges and elongates with age due to material fatigue from cyclic longitudinal stretches and the elongation of the ascending aorta [8,9]. Moreover, the diameter of the ascending aorta and three-dimensional measurements of the aortic arch significantly increase with age [10]. Interestingly, however, the JL5 group was younger than the JL4 group. One possible explanation is that the JL5 group may have included patients with more significant atherosclerosis, as it had a higher proportion of males and smokers. Additionally, the cohort in this study included many patients with severe atherosclerosis, such as severe aortic stenosis (63.1%, 164/260), and an older age profile (median age, 78 years). The relationship between age and dilation or elongation of the ascending aorta in older cohorts remains poorly understood. We believe that the procedure of engaging the catheter in the coronary artery is more influenced by morphological characteristics than patient characteristics. Therefore, we focused on identifying the best predictor for the need for a JL5 coronary diagnostic catheter among the various CT findings of aortic morphology. This is why multivariate analysis was performed solely for aortic morphological findings. We also conducted univariate and multivariate analyses of each significant factor associated with the need for a JL5 in left CAG. The results were largely consistent with those of the nine aortic CT findings (Supplemental material 5).

The JL4 coronary diagnostic catheter was initially designed for the femoral approach [7]. We deemed it necessary to assess each approach site separately due to significant differences in the routes of the guiding coronary catheters for the left coronary artery among the right radial, left radial, and femoral artery approaches [11]. Although we could not identify clear predictors for the femoral artery approach due to the small number of cases, the maximum area of the ascending aorta was a strong predictor for both the left- and right-upper-limb approaches (Supplemental material 5). The cutoff values were 1270.4 mm^2^ (derived diameter, 40.2 mm) for the left-upper-limb approach and 1147.1 mm^2^ (derived diameter, 38.2mm) for the right-upper-limb approach (Supplemental material 3). When using an upper-limb approach, whether right or left, JL5 should be used as the first-attempt diagnostic catheter for engagement in left CAG in cases with large ascending aortas, approximately 38 mm or over.

Some previous studies and trials have demonstrated the usefulness of a single-diagnostic coronary catheter from the radial artery approach, noting benefits such as reduced radial spasm, shorter fluoroscopy times, and lower contrast volume [12-14]. However, Schneider et al. reported that the use of a one-catheter approach in trans-radial diagnostic angiography was inferior to the two-catheter approach in terms of procedure time for younger and male patients [15,16]. The debate whether a one- or two-catheter approach is superior remains unresolved. We believe that the appropriate selection of diagnostic coronary catheters, beyond JR4 and JL4, is crucial and warrants further investigation. Choosing the first-attempt diagnostic catheters for CAG based on aortic morphology from preliminary CT images can potentially optimize the procedure. Since this study only enrolled patients who had CT scans prior to CAG, further studies utilizing other modalities, such as chest X-ray, ECG, and echocardiography, and exploring additional options for appropriate guiding catheter selection in PCI are anticipated.

Limitations

Our present investigation was a single-center retrospective study. Since the type of catheter used and the purpose of the CAG and CT tests were left to the discretion of the operator, operator bias may have influenced the results. For the procedure time, the duration of the right CAG was included because no specific data left CAG time were available. Additionally, the small number of cases in the JL5 group limited the availability to observe severe complications, making it difficult to demonstrate clinical implications in this study. Finally, further investigation, including multicenter, prospective, and randomized studies, is needed to assess clinical practice and patient outcomes in the future.

## Conclusions

The maximum area of the ascending aorta was associated with the need for a larger-curved coronary artery diagnostic catheter (JL5) in left CAG. Using the JL5 as the first diagnostic catheter in patients with an ascending aortic diameter of approximately 38 mm or more could optimize CAG by reducing contrast volume, shortening procedure time, and lowering costs.

## Figures and Tables

**Figure 4:**
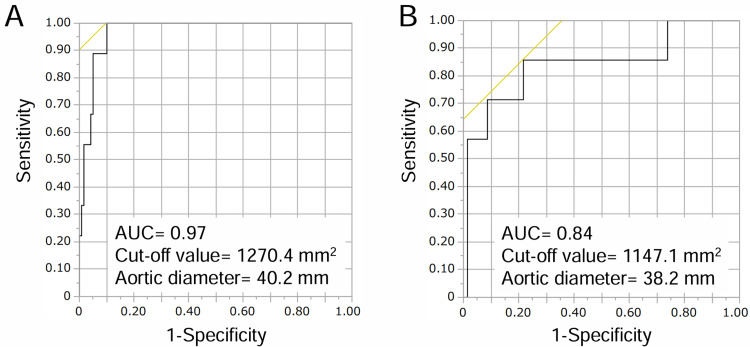
Receiver operating characteristic curve of maximum ascending aorta area for Judkins left 5.0 requirement in left coronary angiography via right-/left-upper-limb approaches (A) Left-upper-limb approach (n = 128); (B) right-upper-limb approach (n = 76).

**Figure 5:**
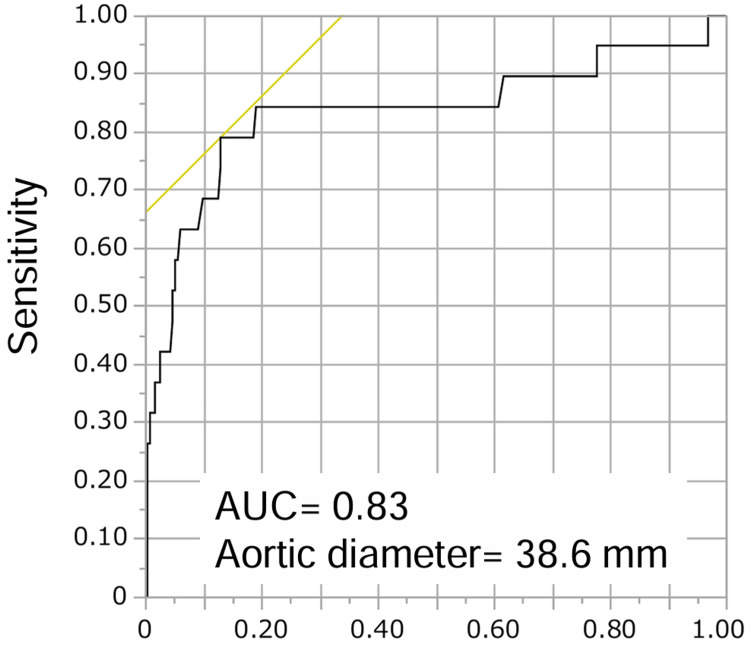
Receiver operating characteristic curve of average aortic diameter for Judkins left 5.0 requirement in left coronary angiography AUC: area under the curve.

## Appendices

Supplemental material 1 

**Table 3:** Reproducibility of the nine aortic morphological findings A: aorta, AA: ascending aorta, LCA: left coronary artery, RCA: right coronary artery.

	Angle of the AA	Angle of the RCA	Angle of the LCA	Maximum area of the AA	Length of the AA	Length of the A	Height of the A	Width of the A	Depth of the A
Intraclass correlation coefficient	0.82	0.90	0.91	0.98	0.94	0.90	0.89	0.91	0.94

Supplemental material 2 

**Table 4:** Differences in aortic morphological findings between the JL5 group and JL4 group in each approach site JL5: Judkins left 5.0, JL4, Judkins 4.0, LCA: left coronary artery, RCA: right coronary artery.

	Total (n = 249)			Left-upper-limb approach (n = 128)			Right-upper-limb approach (n = 76)			Femoral approach (n = 45)		
	JL5 (n = 19)	JL4 (n = 230)	p-value	JL5 (n = 9)	JL4 (n = 119)	p-value	JL5 (n = 7)	JL4 (n = 69)	p-value	JL5 (n = 3)	JL4 (n = 42)	p-value
Angle of the ascending aorta, degrees	48 (46, 54)	49 (42, 55)	0.52	53 (47, 54)	49 (42, 54)	0.34	46 (44, 50)	49 (43, 55)	0.69	48 (48, 58)	48 (41, 53)	0.48
Angle of the RCA, degrees	108 (102, 125)	106 (96, 116)	0.35	116 (98, 125)	106 (96, 114)	0.16	107 (94, 118)	107 (97, 114)	0.95	102 (102, 110)	105 (92, 120)	0.96
Angle of the LCA, degrees	35 (26, 44)	36 (26, 46)	0.74	40 (25, 45)	38 (27, 50)	0.93	28 (21, 35)	33 (22, 45)	0.49	36 (29, 58)	37 (29, 43)	0.80
Maximum area of the ascending aorta, mm^2^	1425 (1179, 1701)	898 (783, 1062)	<0.0001	1493 (1376, 1666)	918 (792, 1054)	0.0001	1646 (1147, 1796)	917 (770, 1114)	0.0030	1111 (568, 1179)	846 (772, 984)	0.37
Length of the ascending aorta, mm	96 (78, 103)	78 (70, 83)	<0.0001	95 (88. 100)	79 (71, 84)	0.0003	97 (71, 105)	78 (71, 83)	0.10	97 (67, 112)	74 (68, 81)	0.20
Total length of the aorta, mm	525 (486, 566)	494 (466, 522)	0.01	516 (494, 540)	497 (469, 531)	0.24	541 (474, 580)	494 (464, 517)	0.05	557 (440, 647)	492 (460, 512)	0.31
Height of the aorta, mm	342 (320, 370)	341 (317. 361)	0.36	341 (329, 368)	342 (320, 362)	0.72	346 (311, 387)	334 (314, 361)	0.80	370 (311, 406)	340 (317, 357)	0.28
Width of the aorta, mm	80 (69, 92)	71 (62, 81)	0.02	82 (74, 92)	71 (62, 82)	0.03	79 (68, 92)	72 (64, 81)	0.18	69 (40, 107)	69 (59, 80)	1.00
Depth of the aorta, mm	122 (115, 140)	115 (105, 124)	0.0035	124 (115, 137)	114 (105, 123)	0.03	120 (115, 138)	116 (105, 126)	0.18	142 (106, 143)	116 (109, 123)	0.21

Supplemental material 3 

Supplemental material 4 

Supplemental material 5 

**Table 5:** Univariate and multivariate analyses of each significant item associated with the requirement for JL5 in left coronary angiography The odds ratios were calculated as the ratio of 10 changes for all items except for the maximum area of the ascending aorta (of 100 changes). CI: confidence interval, LCA: left coronary artery, RCA: right coronary artery.

	Univariate analysis			Multivariate logistic analysis		
	Odds ratio	95% CI	p-value	Odds ratio	95% CI	p-value
Age, year	0.68	(0.48-0.95)	0.03			
Body height, cm	1.81	(1.11-2.96)	0.01			
Body weight, kg	1.71	(1.18-2.49)	0.0041			
Body mass index, kg/m^2^	1.13	(0.99-1.28)	0.06			
Diabetes mellitus, n (%)	0.31	(0.09-1.13)	0.05			
Smoking, n (%)	2.97	(1.09-8.09)	0.03			
Maximum area of the ascending aorta, mm^2^	1.63	(1.30-1.95)	<0.0001	1.75	(1.37-2.22)	<0.0001
Length of ascending aorta, mm	3.09	(1.94-4.90)	<0.0001	2.50	(1.34 – 4.65)	0.0026
Total length of the aorta, mm	1.15	(1.04-1.28)	0.0053			
Width of the aorta, mm	1.39	(1.04-1.88)	0.03	0.47	(0.28-0.79)	0.0026
Depth of the aorta, mm	1.94	(1.32-2.87)	0.0004			
